# Demographic changes in Pleistocene sea turtles were driven by past sea level fluctuations affecting feeding habitat availability

**DOI:** 10.1111/mec.16302

**Published:** 2021-12-14

**Authors:** Jurjan P. van der Zee, Marjolijn J.A. Christianen, Martine Bérubé, Mabel Nava, Sietske van der Wal, Jessica Berkel, Tadzio Bervoets, Melanie Meijer zu Schlochtern, Leontine E. Becking, Per J. Palsbøll

**Affiliations:** ^1^ Marine Evolution and Conservation Groningen Institute for Evolutionary Life Sciences University of Groningen AG Groningen the Netherlands; ^2^ Wageningen Marine Research Den Helder the Netherlands; ^3^ Aquatic Ecology and Water Quality Management Group Wageningen University & Research Wageningen the Netherlands; ^4^ Center for Coastal Studies Provincetown Massachusetts USA; ^5^ Sea Turtle Conservation Bonaire Kralendijk Bonaire Caribbean Netherlands; ^6^ Turtugaruba Foundation Oranjestad Aruba; ^7^ Sint Eustatius National Parks Foundation Sint Eustatius Caribbean Netherlands; ^8^ Sint Maarten Nature Foundation Cole Bay Sint Maarten; ^9^ Dutch Caribbean Nature Alliance Kralendijk Bonaire Caribbean Netherlands; ^10^ Marine Animal Ecology Group Wageningen University & Research Wageningen the Netherlands

**Keywords:** ddRAD sequencing, demographic change, habitat availability, Pleistocene sea turtles, sea level change

## Abstract

Pleistocene environmental changes are generally assumed to have dramatically affected species’ demography via changes in habitat availability, but this is challenging to investigate due to our limited knowledge of how Pleistocene ecosystems changed through time. Here, we tracked changes in shallow marine habitat availability resulting from Pleistocene sea level fluctuations throughout the last glacial cycle (120–14 thousand years ago; kya) and assessed correlations with past changes in genetic diversity inferred from genome‐wide SNPs, obtained via ddRAD sequencing, in Caribbean hawksbill turtles, which feed in coral reefs commonly found in shallow tropical waters. We found sea level regression resulted in an average 75% reduction in shallow marine habitat availability during the last glacial cycle. Changes in shallow marine habitat availability correlated strongly with past changes in hawksbill turtle genetic diversity, which gradually declined to ~1/4th of present‐day levels during the Last Glacial Maximum (LGM; 26–19 kya). Shallow marine habitat availability and genetic diversity rapidly increased after the LGM, signifying a population expansion in response to warming environmental conditions. Our results suggest a positive correlation between Pleistocene environmental changes, habitat availability and species’ demography, and that demographic changes in hawksbill turtles were potentially driven by feeding habitat availability. However, we also identified challenges associated with disentangling the potential environmental drivers of past demographic changes, which highlights the need for integrative approaches. Our conclusions underline the role of habitat availability on species’ demography and biodiversity, and that the consequences of ongoing habitat loss should not be underestimated.

## 1 INTRODUCTION

Environmental changes driven by Pleistocene climate changes were a major driver of past demographic changes (Hewitt, 2000). Global cooling resulted in the formation of continental ice sheets across the northern hemisphere, equator‐ward shifts in temperate climate zones and regressed sea levels during glacial periods (Bintanja et al., 2005). The opposite occurred during interglacial periods: continental ice sheets receded, temperate climate zones shifted towards the poles and sea levels increased. The environmental changes associated with the Pleistocene glacial cycles severely altered the range and distribution of many species; populations recurrently experienced phases of contraction and isolation during glacial events, followed by population expansion and possible secondary contact or admixture between previously isolated populations during interglacial periods (Hewitt, 2000). Consequentially, many natural populations underwent dramatic changes in abundance or became extinct as a result of the environmental changes driven by Pleistocene climate oscillations. Understanding the ecological drivers that underlie the demographic changes in response to past climate change may provide valuable insights in how species can respond to current climate change, akin to a natural experiment on an evolutionary timescale.

Changes in habitat availability have often been invoked as a causal mechanism for the demographic changes associated with Pleistocene climate changes (Dalén et al., 2007; Foote et al., 2013; Hewitt, 2000). For example, declines in Beringian steppe bison populations were attributed to reduced steppe‐tundra habitat and expanding boreal forests, which restricted dispersal and provided poor feeding habitat (Shapiro, 2004). Changes in habitat availability may even drive extinction if species are unable to track these changes. European Arctic fox populations probably became extinct as a result of a failure of tracking changes in habitat after the Last Glacial Maximum (LGM; Dalén et al., 2007). Similar observations have been made in the marine environment, such as signatures of genetic bottlenecks in fishes associated with shallow reef habitats lost due to sea level regression, but not in species inhabiting deeper reef fringes (Fauvelot et al., 2003). These observations result in the general assumption that large‐scale changes in habitat availability regulated species’ abundance, an assertion that can be assessed by tracking past changes in abundance and habitat availability. However, our limited knowledge of Pleistocene ecosystem changes restricts our ability to assess the postulated association between habitat and abundance over extended timescales.

Past sea level fluctuations are well‐mapped (Bintanja et al., 2005) and present an opportunity for assessing the relationship between past abundance and changes in habitat availability driven by Pleistocene climate change. Sea level changes had major impacts on coastal ecosystems that were linked to the availability of shallow marine habitat (Ludt & Rocha, 2015). Consequentially, past sea level changes provide an unique opportunity to assess the relationship between Pleistocene climate changes, habitat availability and past demographic changes. Assessing this relationship can also help provide insights in the long‐term effects of contemporary habitat loss driven by current climate change and other anthropogenic disturbances.

Population genetic theory predicts a relatively straightforward relationship between abundance and genetic diversity in the absence of spatial population substructure for populations at long‐term equilibrium (Hudson et al., 1990). The parameter *θ* = 4*N*_e_ *μ*, where *N*_e_ is the effective population size (Wright, 1931) and *μ* is the per‐generation mutation rate, describes the amount of genetic diversity observed at diploid, autosomal loci (Watterson, 1975). The relationship between genetic diversity and abundance can be leveraged to estimate past demographic changes (e.g., Liu & Fu, 2015). Advances in high‐throughput sequencing now enable generating thousands of single nucleotide polymorphism (SNP) markers at relatively low cost in non‐model species (Ellegren, 2014), providing unprecedented resolution for studying species’ demographic history. Inferred demographic changes can subsequently be linked to ecological changes driven by past climate change to disentangle the underlying processes.

Sea turtles represent an excellent species complex for studying the relationship between past sea level fluctuations, habitat availability and demographic changes because sea turtles typically depend on shallow marine habitat for feeding (Hendrickson, 1980). For example, green turtles (*Chelonia mydas*) are specialized herbivores that graze on seagrass meadows (Bjorndal, 1980) and hawksbill turtles (*Eretmochelys imbricata*) are spongivores associated with feeding in coral reefs (León & Bjorndal, 2002). Bottom‐up regulation is believed to play an important role in regulating sea turtle population dynamics (Jackson, 1997), suggesting changes in feeding habitat availability could lead to concurrent changes in abundance. As mentioned earlier, relating past changes in genetic diversity to abundance requires spatial substructure to be absent (Hudson et al., 1990; Watterson, 1975). Genetic differentiation estimated using nuclear markers is typically reduced due to a high degree of male‐mediated gene flow common in sea turtles (Roberts et al., 2004). Changes in genetic diversity estimated from nuclear markers are therefore expected to be an appropriate proxy for changes in abundance in sea turtles. Furthermore, coastal ecosystems, such as seagrass meadows and coral reefs, are highly threatened by climate change and anthropogenic disturbances (Jackson et al., 2001; Waycott et al., 2009). The potential loss of key feeding habitat is of major concern to sea turtle conservation. Studying how sea turtle populations responded to past changes in feeding habitat availability can aid predicting the consequences of contemporary feeding habitat loss.

Here, we evaluated the role of past changes in habitat availability driven by Pleistocene climate changes as a mechanism driving demographic changes by assessing changes in shallow marine habitat availability (0–60 m deep) during the last 125 thousand years (kya) in the wider Caribbean. We evaluated the association between changes in shallow marine habitat availability and changes in genetic diversity of the hawksbill turtle inferred from genome‐wide single nucleotide polymorphism (SNP) markers. The hawksbill turtle is a migratory tropical marine vertebrate closely associated with tropical coral reefs, where they fulfill a keystone role in structuring sponge communities in coral reefs in the Caribbean (León & Bjorndal, 2002). The majority of coral reefs are located in shallow (<40 m) tropical waters (Huston, 1985; Kleypas et al., 1999), suggesting that coral reef habitat availability was severely reduced during periods of lowered sea levels. The Caribbean represents an excellent study area, since shallow marine habitat was reduced to <10% of the present‐day area during the LGM (Ludt & Rocha, 2015). However, we have no insights into how sea level changes affected shallow marine habitat availability during the entire last glacial cycle.

## 2 MATERIALS AND METHODS

### 2.1 Sample collection and DNA extraction

To obtain a representative sample of Caribbean‐wide nuclear genetic diversity, we sampled individuals at different life stages and at multiple nesting beaches and feeding grounds in the northern and southern Caribbean (Table S1). Hatchling were sampled by excising a small piece of the flipper from stillborn individuals encountered during post‐hatching nest excavations. Juveniles were hand‐captured by scuba/snorkel‐assisted free‐divers. Adults were sampled post‐nesting. A small sliver of skin tissue was excised from the dorsal neck epidermal area using a sterilized scalpel blade. Samples were preserved and stored in 6 M sodium chloride and with 25% dimethyl sulphoxide (Amos & Hoelzel, 1991). Total‐cell DNA was extracted using the Gentra Puragene Tissue Kit (Qiagen Inc.) following the manufacturer's instructions and resuspended in 1x TE (10 mM Tris‐HCl, 1mM EDTA, pH 8.0). The average size and quality of the extracted DNA was assessed by gel electrophoresis through a 0.7% agarose (AMRESCO Inc.) gel for 20 minutes at 175V and 500 mA. The DNA was stained with ethidium bromide and visualized under UV light. The quantity of DNA was estimated using a Qubit Fluorometer (Life Technologies) following the manufacturer's instructions. An aliquot of each DNA extraction was normalized with 1×TE to a final concentration at 20 ng/μl.

### 2.2 Library preparation

A single next‐generation sequencing library was prepared following the original ddRAD protocol (Peterson et al., 2012), though we included a modification based upon the quaddRAD protocol (Franchini et al., 2017). Genomic DNA was digested using *HindIII* and *mspI* restriction enzymes. Adapters were designed with overhangs compatible with our restriction enzymes and a random stretch (NNNN) to identify and bioinformatically remove PCR duplicates (Franchini et al., 2017). The ligated DNA with barcodes were pooled and was size‐selected at a 300–400 bp range using a Pippin Prep (Sage Science Inc.). The fragments were then enriched and uniquely indexed using a Phusion polymerase kit (New England Biolabs). The final library was diluted to a concentration of 1.97 ng/μl in a volume of 20 μl. The library was sequenced on an Illumina HiSeq4000 as paired‐end in high‐throughput mode at Novogene Company Ltd. in Hong Kong.

### 2.3 Cleaning, demultiplexing and read mapping

Raw sequence reads were demultiplexed and cleaned using process_radtags in STACKS version 1.47 (Catchen et al., 2013). Samples with fewer than 1.5 million reads were excluded. Paired‐end reads were mapped to the green turtle reference genome (Wang et al., 2013) with bowtie2 version 2.3.3.1 (Langmead et al., 2009) using “very‐sensitive” and “end‐to‐end” alignment. The paired‐end size restriction was set to the default settings. Discordant reads were aligned as unpaired reads.

### 2.4 Population structure, inbreeding and kinship

SNPs were called from mapped reads using the marukilow model (Maruki & Lynch, 2017) as implemented in gstacks in stacks version 2.2 (Catchen et al., 2013). All samples were treated as originating from the same population (“DC”; Dutch Caribbean). We only included SNPs genotyped in at least 80% of all samples (Paris et al., 2017). SNPs with a heterozygosity above 0.5 were excluded. SNPs within less than 10,000 base pairs (bp) distance from other SNPs were removed using vcftools version 0.1.16 (Danecek et al., 2011) to reduce physical linkage among SNPs. To assess the selective neutrality of our data, we performed variant annotation and effect prediction of SNPs using snpeff version 4.3 t (Cingolani et al., 2012).

Because population structure can confound demographic inference (Chikhi et al., 2010), we assessed the presence of population genetic structuring among the samples using model‐based clustering and multivariate approaches. We conducted model‐based clustering with faststructure version 1.0 (Raj et al., 2014) using a simple prior to identify the optimal number of clusters describing our data. Multivariate‐based clustering and principal component analyses were conducted using the adegenet R package version 2.1.3 (Jombart, 2008; Jombart & Ahmed, 2011) in r version 4.0.4 (R Core Team, 2021). We estimated pairwise relatedness using the KING‐robust method (Manichaikul et al., 2010) implemented in snprelate version 1.16.0 (Zheng et al., 2012) in r version 4.0.4.

### 2.5 Past demographic changes

Past demographic changes were inferred using genotype likelihoods, which has been shown to lead to more robust demographic inference (Warmuth & Ellegren, 2019). The folded site frequency spectrum (SFS) was estimated from genotype likelihoods (Nielsen et al., 2012) using angsd version 0.925 (Korneliussen et al., 2014). Genotype likelihoods were estimated from the mapped reads (minimum mapping quality of 10 and minimum read quality of 20) with angsd version 0.925 (Korneliussen et al., 2014) using the SAMTOOLS method. Given that we employed a probabilistic framework, i.e. using genotype likelihoods, we applied a less stringent filter where sites not present in at least 50% of individuals were excluded. The minimum depth per site was set to the default value. The folded SFS was used because the derived allele could not be reliably determined due to the evolutionary distance between the hawksbill‐, and green turtle (i.e. *t*_MRCA_ ≈ 55 million years ago; Duchêne et al., 2012). Past changes in *θ* were estimated from the folded SFS using the stairway plot method (Liu & Fu, 2015) assuming a mutation rate at 7.9 × 10^−9^ substitutions per site per generation estimated for crocodilians (Green et al., 2014) and a generation time at 35 years (Meylan & Donnelly, 1999). Because crocodilians probably have lower mutations rates than turtles (Green et al., 2014; Shaffer et al., 1997), we also investigated the effects of a higher mutation rate at 1.2 × 10^−8^ substitutions per site per generation (Kong et al., 2012) previously applied to demographic inferences in green turtles (Fitak & Johnsen, 2018).

### 2.6 Past changes in shallow marine habitat availability

Modern (Sbrocco & Barber, 2013) and LGM (21 kya) bathymetry and sea surface temperature data (Braconnot et al., 2007; Sbrocco, 2014) was used to map Caribbean shallow marine habitat availability, defined as grid points with a depth between 0 and 60 m (Ludt & Rocha, 2015) and a minimum mean sea surface temperature of >20°C based upon the thermal limits of hawksbill turtles (Davenport, 1997), at a 5 arc‐minute resolution. We also estimated modern and LGM shallow marine habitat availability for the Southwest Atlantic (55°W–30°W; 35°S–2°S) and East Atlantic regions (30°W–15°E; 5°S–20°N), encompassing extant Brazilian (e.g., Proietti et al., 2014) and West African (e.g., Monzón‐Argüello et al., 2011) hawksbill turtle breeding and feeding grounds. Past sea levels (Bintanja et al., 2005) were used to estimate past changes in shallow marine habitat availability between 125–0 kya by calculating the number of grid points in the Caribbean (Figure S1 for details) with a depth between 0 + *S* and 60 + *S* m, where *S* was the sea level anomaly. To estimate past changes in shallow marine habitat availability in the Caribbean, we assumed sea surface temperatures (SSTs) did not restrict feeding habitat availability because tropical SSTs were only marginally lower during the LGM (Herbert et al., 2010) and well above hawksbill turtle thermal limits (Davenport, 1997). The association between genetic diversity (measured as median *θ*) and past changes in shallow marine habitat availability (relative to the present), as well as mean global surface air temperature and sea level anomalies (Bintanja et al., 2005) was assessed via Pearson correlation using r version 4.0.4.

To account for the time‐series nature of the data, we additionally performed a regression analysis using generalized least squares with the nlme R package (version 3.1–152; r version 4.0.4), where an autoregressive process of order 1 was used to model autocorrelation among residuals. We initially included shallow marine habitat availability, global surface air temperature and sea levels as predictors of past changes in genetic diversity, and subsequently explored simpler models by excluding predictors. The models were fitted by maximizing the restricted log‐likelihood. Model performance was evaluated using the Aikaike information criterion (AIC) and Bayesian information criterion (BIC).

## 3 RESULTS

We generated genotyping by sequencing data for *N* = 55 hawksbill turtles sampled at different life stages and at multiple nesting beaches and feeding grounds in the northern and southern Caribbean (Table S1). The mean number of reads per sample was approximately 10.7 × 10^6^ (range: 1.0 × 10^6^ and 33.0 × 10^6^). The mean alignment rate against a green turtle draft genome (Wang et al., 2013) was 89% (Table S1). A single sample with few reads (2.2 × 10^5^) and an alignment rate at only 37.5% was excluded from further analysis. We additionally excluded another sample with missing genotypes at more than 50% of the identified SNPs (Figure S2). The remaining 53 samples were genotyped at a total of 25,732 loci. Thinning SNPs to ensure a minimum distance of 10,000 base pairs among SNPs resulted in a final data set comprising 14,861 SNPs genotyped at rates of 80% or above. The majority of SNPs (~98.6%) were associated with non‐coding genomic regions (Table S2). Predicted functional impacts classified ~99.9% of the SNPs as either non‐coding variants, variants of non‐coding genes or showing no evidence of functional impacts (Table S2). A few SNPs were found in splice site regions, but were predicted to unlikely affect protein functionality (Table S2).

**FIGURE 1:**
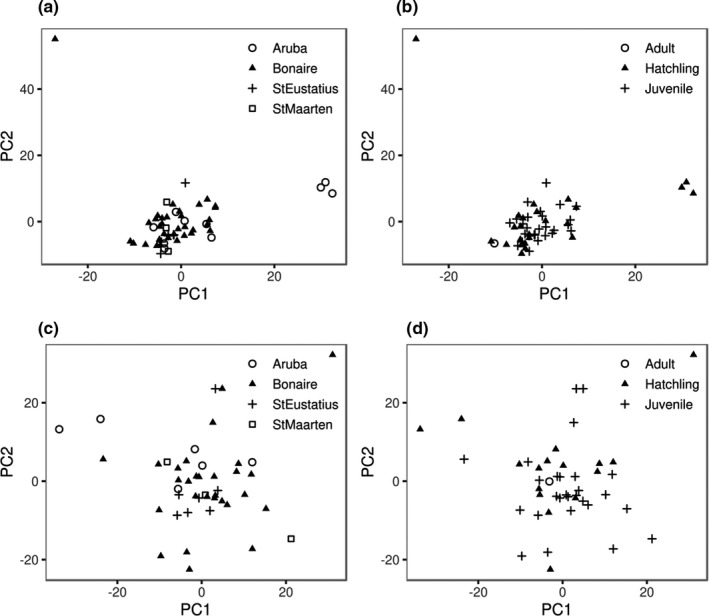
Plot of the first two principal components with individuals labelled by (a,c) sampling location (b,d) and sample type estimated from the (top panels; a–b) data including putative related individuals (*N* = 53) and (bottom panels; c–d) data excluding putative related individuals (*N* = 44)

Model‐based and multivariate‐based clustering showed our data was best described by a single cluster (Table S3; Figure S3). A principal component analysis suggested no samples clustered according to geography (Figure 1a). Variation along the first principal component axis revealed a distinct cluster (Figure 1b) comprised of three hatchling samples (AR150002, AR150005 and AR150008; Figure S4). Variation along the second principal component axis additionally revealed a single outlier sample (BO160241; Figure 1a–b), which was a hatchling sampled in Bonaire (Figure S4; Table S1). Most of the relevant structure in the data appeared to be captured by the first two principal components (Figure S5; Jombart et al., 2009). Pairwise estimates of kinship coefficients ranged between 0.16 and 0.24 (Figure S6) for eight dyads, suggesting that our sample contained putative sibling‐, and parent‐offspring pairs (Weir et al., 2006). No other dyads were found in the data set. Randomly excluding one individual from dyads (and two from one triad) of putative related individuals, respectively, as well as the outlier sample BO160241 resulted in a single cluster comprised of *N* = 44 samples (Figure 1c–d; Figure S7). A lack of structure was additionally suggested by a gradual decline in inertia (Figure S8; Jombart et al., 2009) and by model‐based clustering (Table S3).

**FIGURE 2:**
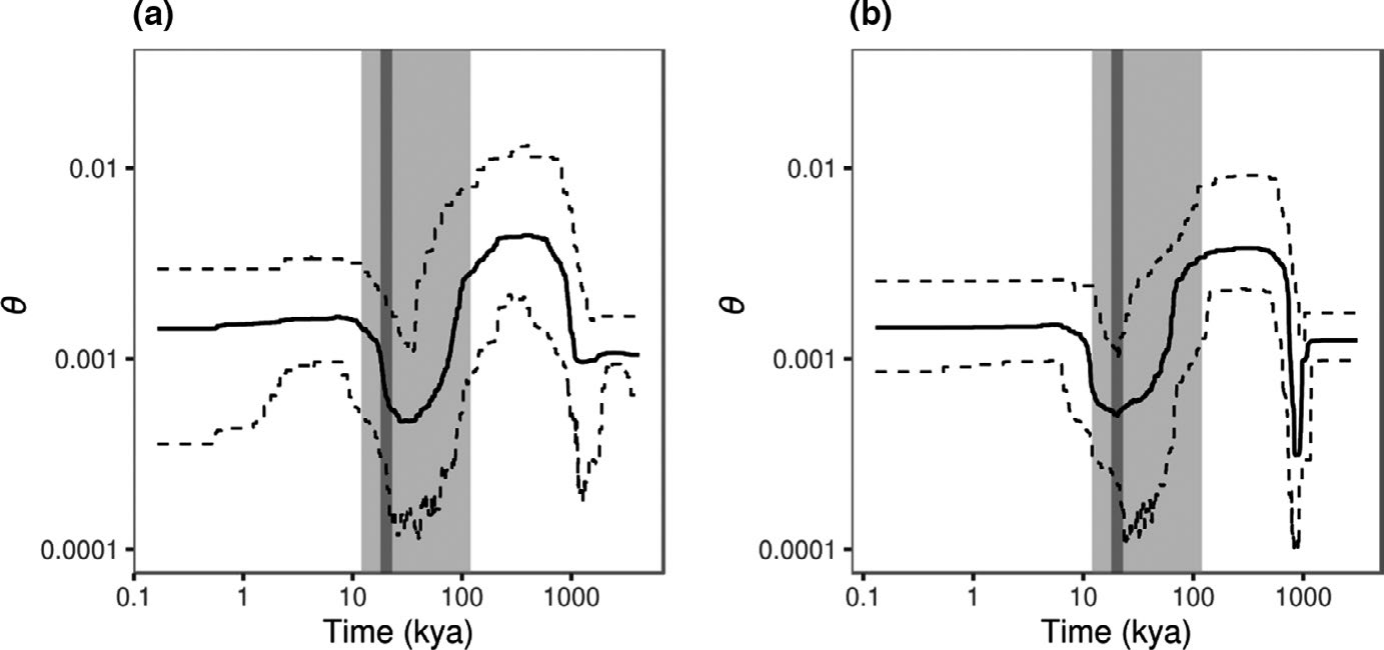
Median (solid line) and 95% confidence interval (dashed lines) of genetic diversity (*θ*) through time (kya) estimated from the data excluding putative related individuals (*N* = 44 individuals). Results are shown for (a) *μ* = 7.9 × 10^−9^ and (b) *μ* = 1.2 × 10^−8^ substitutions per site per generation. The approximate timing of the last glacial cycle (120–14 kya) is indicated in light grey shading. The LGM (26–19 kya) is indicated by a dark grey shaded bar

To account for a potential effect of population structure, demographic inference was performed on the reduced *N* = 44 data set. Past changes in genetic diversity estimated from the folded site frequency spectrum (SFS; Figures S9–S10) revealed a decline in genetic diversity (Figure 2) during the last glacial period (120–14 kya) followed by an increase after the LGM (26–19 kya). Applying a lower mutation rate resulted in older time points (Figure 2a), whereas applying a higher mutation rate had the opposite effect (Figure 2b). Analyses including putative related individuals resulted in more recent demographic changes (Figure S11). In addition, two decline and expansion cycles were observed for the higher mutation rate (Figure S11B), possibly an effect of population substructure due to inclusion of putative related individuals.

**FIGURE 3:**
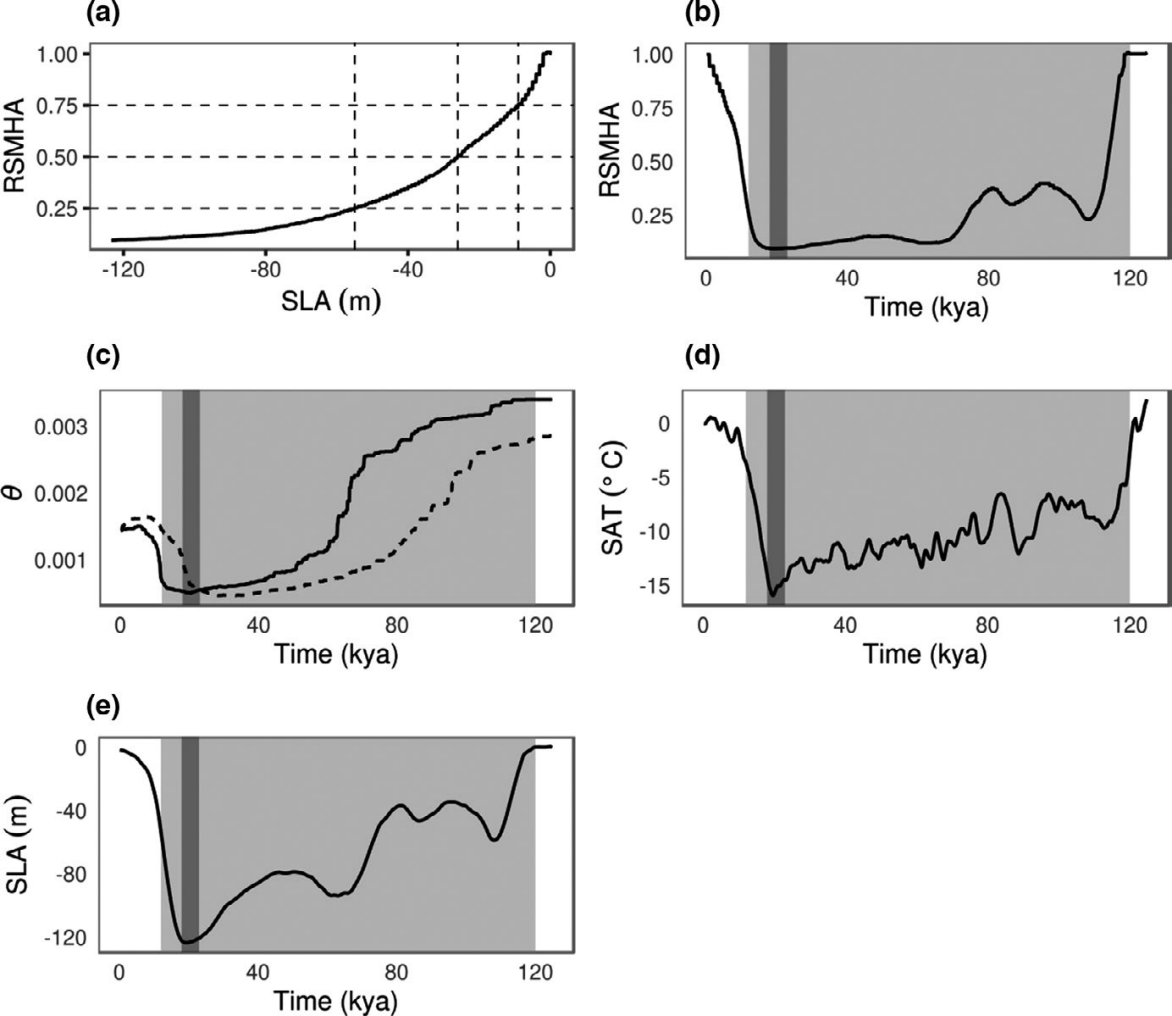
Shallow marine habitat availability relative to the present (RSMHA) versus (a) the mean global sea level anomaly (SLA) in metres (m), and past trajectories of (b) RSMHA, (c) median genetic diversity (*θ*) for *μ* = 7.9 × 10^−9^ (dashed line) and *μ* = 1.2 × 10^−8^ (solid line) substitutions per site per generation, (d) the mean global surface air temperature anomaly (SAT) in degrees Celsius (°C) and (e) the SLA during the last 125 thousand years. The approximate timing of the last glacial cycle (120–14 kya) is indicated in light gray shading. The LGM (26–19 kya) is indicated by a dark grey shaded bar

Relatively minor decreases in sea levels resulted in considerable declines in shallow marine habitat availability; a 25%, 50% and 75% reduction in shallow marine habitat availability was achieved at sea levels 9, 26 and 55 m lower than present‐day levels, respectively (Figure 3a). Shallow marine habitat availability was estimated at 9.6% of present‐day levels in the Caribbean during the LGM (Figure S12; Table S4). Caribbean mean annual sea surface temperatures were 2 to 3°C lower during the LGM, ranging between 24 and 26°C (Figure S12). Shallow marine habitat availability in the Southwest‐, and East Atlantic regions was estimated at 15.7% and 6.8% during the LGM, respectively (Table S4). Southwest‐, and East Atlantic LGM shallow marine habitat availability declined to 19.2% and 7.4%, respectively, when no minimum sea surface temperature of 20°C was assumed (Table S4). We estimated that the availability of Caribbean shallow marine habitat declined to <25% (relative to the present) during the early stage of the last glacial cycle (120–110 kya), increasing to 35%–40% (110–80 kya) followed by a decline to <10% (80–19 kya; Figure 3b). The mean availability of shallow marine habitat during the period 120–14 kya was 75% lower than present‐day levels. Past changes in shallow marine habitat availability (Figure 3b) and sea levels (Figure 3E) correlated strongly (Table S5) with genetic diversity (Figure 3c). Global surface air temperatures (Figure 3d) showed a weaker correlation with genetic diversity (Table S5). The AIC, BIC and log‐likelihoods consistently supported a simple model that involved a single predictor variable in the regression analysis, but model rankings differed between mutation rates (Table S6). Past changes in genetic diversity were best predicted by either global surface air temperatures (*μ* = 7.9 × 10^−9^ substitutions per site per generation) or sea level changes (*μ* = 1.2 × 10^−8^ substitutions per site per generation), while the model including shallow marine habitat availability ranked third and second, respectively.

## 4 DISCUSSION

In the present study, the role of past changes in habitat availability driven by Pleistocene climate changes as a potential driver of past demographic changes was investigated in Caribbean hawksbill turtles. We showed that Pleistocene sea level regression severely reduced shallow marine habitat availability in the Caribbean during the last glacial cycle (120–14 kya) and detected concordant declines in the genetic diversity of hawksbill turtles. Shallow marine habitat availability and genetic diversity showed a synchronous and rapid increase to present‐day levels after the LGM. However, sea level changes and surface air temperatures also showed strong correlations with Caribbean hawksbill genetic diversity, and we found no consistent support for a single driver of past demographic changes. In part, these findings can be attributed to non‐independence among the investigated drivers, i.e., temperature is indirectly related to sea levels via the formation and melting of continental ice sheets, and in turn to shallow marine habitat availability. Temperature probably also influenced other abiotic or biotic processes, e.g., the distribution and quality of nesting habitat for sea turtles (Pike, 2013), sex ratios (Davenport, 1997) and sea turtle growth rates (Bjorndal et al., 2017), factors that we did not consider. It did not appear, however, that sea surface temperatures limited the distribution of Caribbean hawksbill turtles. Tropical sea surface temperatures were on average 2 to 3°C lower during glacial maxima (Herbert et al., 2010) and ranged between 24 and 26°C in the Caribbean during the LGM. These sea surface temperatures fall within the range (19–30°C) experienced at present‐day hawksbill turtle feeding grounds (Diez & van Dam, 2002; Gaos et al., 2012). Our findings thus potentially suggest that Pleistocene sea level changes affected the distribution and quantity of feeding habitat, and subsequently caused demographic changes in Caribbean hawksbill turtles. Similar observations have been made in marine vertebrates with different temperature tolerances (Foote et al., 2013; Morin et al., 2015). In the arctic‐adapted bowhead whale (*Balaena mysticetus*), a post‐LGM population expansion was concordant with a threefold increase in suitable habitat area following the retreat of permanent sea ice in the northern Atlantic (Foote et al., 2013). By contrast, killer whales (*Orcinus orca*) showed no evidence of past demographic changes, consistent with relatively minor declines (15%) in suitable habitat availability during the LGM concordant with their broad distribution between the tropics and the arctic regions (Morin et al., 2015). In other words, large‐scale changes in the availability of feeding habitat could have affected the population dynamics of species dependent upon this habitat in a bottom‐up manner, which has also been suggested for green turtles (Jackson, 1997). However, we cannot rule out potential influences from other environmental factors, and the impact of Pleistocene climate change on other aspects of the life history of hawksbill turtles warrants further exploration.

An additional complication is that changes in genetic diversity are typically expected to lag behind changes in abundance (Palsbøll et al., 2013), which has implications for evaluating the environmental drivers of past demographic changes. For example, simulated populations subjected to an instantaneous 95% bottleneck (i.e., a decline from *θ* = 6.0 to *θ* = 0.3) showed a 7% and 50% decrease in genetic diversity after 200 and 2000 generations, respectively (Palsbøll et al., 2013). In other words, a gradual decline in genetic diversity is expected after a rapid decline in abundance. As a result, a strong correlation of environmental drivers (e.g., sea levels or temperature) with past changes in genetic diversity does not necessarily imply a similar relationship with abundance, the parameter of interest. Integrative approaches (Hoban et al., 2019) are ultimately required to disentangle the abiotic and biotic drivers of past demographic changes, where past and present habitat availability is modeled explicitly using species distribution and environmental data (Alvarado‐Serrano & Knowles, 2014). Such models could also serve as input for spatially‐explicit coalescent‐based assessments of genetic data (Ray et al., 2010), enabling model selection to further evaluate the factors underpinning the demographic response to Pleistocene climate changes.

The relationship between past environmental fluctuations and demographic change can potentially be further obscured by adaptive responses. For example, it is possible that hawksbill turtles adapted to reduced shallow marine habitat availability by shifting towards feeding in deeper habitats, given that hawksbill turtles may dive up to 60 m (Blumenthal et al., 2009). A similar adaptive response was hypothesized for reef fishes (Fauvelot et al., 2003). Species associated with shallow reef habitats shifted to reef fringes, which resulted in a bottleneck but allowed populations to persist through sea level changes (Fauvelot et al., 2003). Declines in abundance are expected to be ameliorated if hawksbill turtles adapted to reduced shallow marine habitat availability by altering their feeding behavior, resulting in a less severe reduction in genetic diversity compared to shallow marine habitat availability, which could explain a >90% reduction in shallow marine habitat availability during the majority of the last glacial period (i.e., 70–19 kya) seemed to be associated with a 75% decline in genetic diversity, relative to present‐day levels. The implicit assumption that an organism's niche stays the same over evolutionary timescales represents a limiting factor when evaluating potential drivers of past demographic chance, but could potentially also be incorporated in an integrative simulation framework.

Previous studies suggested the occurrence of a population decline followed by an expansion in wider Caribbean hawksbill turtles around 900 kya (Reece et al., 2005) and 100–300 kya (Leroux et al., 2012) based upon mitochondrial DNA. The difference between these estimates was attributed to the analysis being performed on individual clades (Leroux et al., 2012) rather than on the pooled sample (Reece et al., 2005) by Leroux and colleagues (2012). By contrast, our results suggested a more recent population decline and expansion associated with the timing of the last glacial cycle, consistent with post‐LGM population expansions reported for Indo‐Pacific hawksbill turtles (Vargas et al., 2016) and green turtles (Reid et al., 2019). The differences between our findings and the studies of Reece et al. (2005) and Leroux et al. (2012) might be attributed to two causes. First, the large number of SNP markers employed in our study provides enhanced resolution compared to single‐marker‐based approaches, allowing for detailed reconstruction of past demographic histories (Edwards & Beerli, 2000). Second, natal philopatry drives strong geographic structuring of mitochondrial DNA variation in sea turtles (Bass et al., 1996; Bowen et al., 1992; Jensen et al., 2019; Leroux et al., 2012; Vargas et al., 2016) due to the maternal inheritance of mitochondria (Birky et al., 1989) and consequently probably confounds demographic inferences based upon mitochondrial markers (Heller et al., 2013; Hudson et al., 1990). By contrast, we applied nuclear markers and carefully assessed whether population substructure was present using both model‐based and multivariate approaches, which suggested our sample of Caribbean hawksbill turtles behaved as a single panmictic population. These findings are consistent with the typically low levels of genetic differentiation estimated from nuclear markers due to elevated male‐mediated gene flow in sea turtles (Roberts et al., 2004), highlighting the value of nuclear markers for studying the demographic history of sea turtles.

If intraoceanic gene flow (at nuclear loci) is sufficiently high, it is possible that the estimated past changes in genetic diversity reflect demographic changes across larger spatial scales. Atlantic hawksbill turtle nesting populations are located in the Caribbean (Bass et al., 1996; Bowen et al., 2007; Leroux et al., 2012), in the Southwest Atlantic (i.e., Brazil, e.g., Proietti et al., 2014) and the East Atlantic (i.e., West and Central Africa, e.g., Monzón‐Argüello et al., 2011). The Caribbean accounts for most nesting activity with ~3,000 adult females nesting annually, compared to ~470 in Brazil and <10 in West and Central Africa (Mortimer & Donnelly, 2008). Long‐distance juvenile dispersal among the Caribbean, Southwest‐, and East Atlantic regions has been demonstrated by tagging and genetic analysis (Bowen et al., 2007; Proietti et al., 2014; Santos et al., 2019). For example, a juvenile hawksbill turtle recorded in the US Virgin Islands contained a haplotype of the mitochondrial DNA control region (Bowen et al., 2007) only described in East Atlantic nesting populations (Monzón‐Argüello et al., 2011). However, a preference for feeding closer to natal regions has been suggested for larger juveniles (Bowen et al., 2007) and migration distances are lower in adults (327 ± 387 km; mean ± SD) compared to juveniles (2675 ± 3212 km; mean ±SD; Hays & Scott, 2013). Post‐nesting movements of adult Caribbean hawksbill turtles suggest they primarily migrate to feeding areas within the Caribbean, with maximum reported straight‐line distances of up to 1600–1700 km (Becking et al., 2016; Van Dam et al., 2008) and females traveling farther than males (Van Dam et al., 2008). Furthermore, significant genetic divergence (*F*_ST_ = 0.033) at microsatellite loci and model‐based clustering suggested reduced gene flow between Caribbean and Southwest Atlantic green turtle nesting populations (Naro‐Maciel et al., 2014). Gene flow among the Caribbean, Southwest‐, and East Atlantic regions might therefore be limited in hawksbill turtles as well. However, occasional gene flow cannot be ruled out; a hatchling sampled in Bonaire (i.e., sample BO160241) appeared to be genetically distinct and might be explained by recent migrant ancestry. Without nuclear genomic data from other regions, however, the spatial scale of panmixia in Atlantic hawksbill turtles unfortunately remains unclear. However, declines in shallow marine habitat availability throughout the tropical Atlantic reflected those observed in the Caribbean, implying a similar demographic response.

The multivariate analysis of genetic variation suggested some heterogeneity within our sample, which could be partially attributed to the presence of related individuals within our sample. Including related individuals may inflate the number of intermediate variants in site frequency spectra, mimicking the effect of population substructure, which in turn can be misinterpreted as evidence of population bottlenecks (Heller et al., 2013; Mazet et al., 2016). However, excluding related individuals may also bias demographic inference (Waples & Anderson, 2017). Accordingly, we conducted demographic inference using different subsets of the data. The timing and magnitude of past changes in genetic diversity appeared consistent across subsets, suggesting there was no apparent bias due to the presence of related individuals. By contrast, past changes in genetic diversity estimated from the full data suggested Caribbean hawksbill turtles recovered slightly during the last glacial period. We cannot reject the possibility the observed partial population recovery is a spurious signal caused by within‐sample substructure due to the presence of related individuals.

Estimated kinship coefficients suggested the observed pairs of related individuals represented putative sibling‐, and parent‐offspring pairs (Weir et al., 2006). Non‐random sampling partially explains the presence of putative related individuals; our sample comprised hatchlings collected from the same nesting site within a single breeding season, as well as one adult. Adult female hawksbill turtles lay multiple nests per breeding season (Richardson et al., 1999), which given the small annual number of nesting females in the Dutch Caribbean (Dow & Eckert, 2011) can lead to a high probability of sampling nests from the same female. The detection of putative siblings and parent‐offspring pairs in an apparent random sample drawn from various nesting beaches and feeding grounds suggests there are opportunities for relatedness‐based inference of population connectivity and abundance (Bravington et al., 2016; Feutry et al., 2017), in sea turtles. For example, close‐kin mark‐recapture applied to the speartooth shark (*Glyphis glyphis*) demonstrated a high degree of juvenile fidelity to river systems (Feutry et al., 2017). In sea turtles, natal philopatry has been demonstrated in adult females and males (FitzSimmons et al., 1997), and to a lesser degree in juveniles (Naro‐Maciel et al., 2017). In our study, two putative sibling‐pairs were comprised of a hatchling and a juvenile. All four individuals were sampled in Bonaire in 2016, which suggested that both juveniles in the two hatchling‐juvenile sibling pairs recruited to feeding areas located in the vicinity of natal nesting sites. These findings imply a direct observation of juvenile natal homing in hawksbill turtles and are consistent with a juvenile preference for feeding areas close to natal regions suggested for hawksbill turtles (Bowen et al., 2007) and other sea turtle species (Bowen et al., 2004).

The genetic diversity of hawksbill turtles prior to the last glacial period was higher than contemporary genetic diversity. Immigration may introduce novel genetic diversity into a population, which, over long time scales implies that local levels of genetic diversity converges towards “global” species genetic diversity (Hudson et al., 1990). The older parts of our estimation of genetic diversity might therefore been inflated by immigration (Beerli & Felsenstein, 1999; Hudson et al., 1990; Palsbøll et al., 2013). The cold Benguela Current is presumed to constitute a biogeographic barrier between the Atlantic and Indian Ocean for tropical marine species, though warm‐water gyres of the Agulhas Current have been suggested to facilitate west‐ward tropical dispersal (Hutchings et al., 2009; Rocha et al., 2005). Warmer conditions prevailed in the Benguela during the early Holocene warm period (Zhao et al., 2017), and sea surface isotherms and sea ice extent shifted northwards in the South Atlantic during the LGM (Gersonde et al., 2003). These changes in large‐scale oceanographic conditions suggest that the permeability of the Benguela Current biogeographic barrier may fluctuate between glacial‐, and interglacial periods. The previous interglacial period (130–116 kya) was warmer than the present. It is possible that the Benguela Current biogeographic barrier was sufficiently weakened during this period to allow a tropical migratory corridor to exist between the Atlantic and Indian Ocean, which has been suggested for green turtles (van der Zee et al., 2021). It is possible that this resulted in increased gene flow between Atlantic and Indo‐Pacific hawksbill turtles, and our elevated estimate of pre‐glacial genetic diversity thus captures a signature of past gene flow.

Due to the uncertainty in mutation rates, we investigated the timing of events using both the slower mutation rate estimated from crocodilians (Green et al., 2014), and a faster mutation rate, similar to the human rate (Kong et al., 2012). The true mutation rate in sea turtles is probably more similar to crocodilians than humans (Green et al., 2014; Shaffer et al., 2013), suggesting the lower mutation rate was more appropriate. Nonetheless, our findings consistently suggested a population decline associated with the timing of the last glacial period.

Despite recent heavy human exploitation of hawksbill turtles and severe habitat degradation (McClenachan et al., 2006), we did not detect any recent decline in genetic diversity as expected during a bottleneck. However, very recent population declines are challenging to detect from genetic data unless a bottleneck was extremely severe, e.g. a reduction to <50 individuals (Peery et al., 2012). The estimate of the recent decline is ~80% decline during three generations (Meylan & Donnelly, 1999) and the current abundance has been estimated at 27,000 adult hawksbill turtles (Bjorndal & Jackson, 2003); a decline that is unlikely to be detectable in genetic data.

The relationship between Pleistocene climate changes, habitat availability and demographic change underlines that the consequences of contemporary habitat loss should not be underestimated. Tropical marine ecosystems have degraded considerably during the most recent decades as a result of human disturbances and climate change (Alvarez‐Filip et al., 2009; Jackson et al., 2001; Waycott et al., 2009). While coral reefs persisted throughout the Pleistocene (Tager et al., 2010), current warming rates are unprecedented and have already impacted coral reefs considerably (Greenstein & Pandolfi, 2008). Certain coral reefs may become sponge reefs in the near‐future (Bell et al., 2013), but it is unclear whether species that are becoming more dominant will be palatable to hawksbill turtles, or whether hawksbill turtles might adapt to ecological changes by targeting other resources in order to adapt to climate change (Bell, 2013).

## CONFLICT OF INTEREST

The authors declare no conflict of interest.

## AUTHOR CONTRIBUTIONS

Jurjan P. van der Zee conceived the study and wrote the manuscript with input from all authors. Per J. Palsbøll supervised the study. Laboratory work was done by Martine Bérubé. Jurjan P. van der Zee conducted bioinformatic‐, and data analyses. Mabel Nava, Leontine E. Becking, Marjolijn J.A. Christianen and Per J. Palsbøll obtained funding. Mabel Nava, Jessica Berkel, Sietske van der Wal, Melanie Meijer zu Schlochtern and Tadzio Bervoets provided tissue samples and logistic support in the field.

## Supporting information

Supplementary Material

## Data Availability

Raw sequencing data has been deposited in Dryad (https://doi.org/10.5061/dryad.7d7wm37wm).
