# Supplementary material for: Demographic changes in Pleistocene sea turtles were driven by past sea level fluctuations affecting feeding habitat availability
Source: Mol Ecol. 2021 Dec 14;31(4):1044–56. doi: 10.1111/mec.16302 (PMC9299637; doi:10.1111/mec.16302)
Supplement: Supplementary file 1 — Supplementary Material [file MEC-31-1044-s001.docx]

**Supplemental Information for:**

**Demographic changes in Pleistocene sea turtles were driven by past sea level fluctuations affecting feeding habitat availability**

Jurjan P. van der Zee, Marjolijn J.A. Christianen, Mabel Nava, Sietske van der Wal, Jessica Berkel, Tadzio Bervoets, Melanie Meijer zu Schlochtern, Martine Bérubé, Leontine E. Becking, Per J. Palsbøll

**Table of Contents:**

| **Title, authors, ToC** | Page 1 |
| --- | --- |
| **Figure S1** | Page 2 |
| **Figures S2-S3** | Page 3 |
| **Figures S4-S5** | Page 4 |
| **Figure S6** | Page 5 |
| **Figures S7-S8** | Page 6 |
| **Figures S9-S10** | Page 7 |
| **Figure S11** | Page 8 |
| **Figure S12** | Page 9 |
| **Table S1 (part 1)** | Page 10 |
| **Table S1 (part 2)** | Page 11 |
| **Tables S2-S3** | Page 12 |
| **Tables S4-S5** | Page 13 |
| **Table S6** | Page 14 |


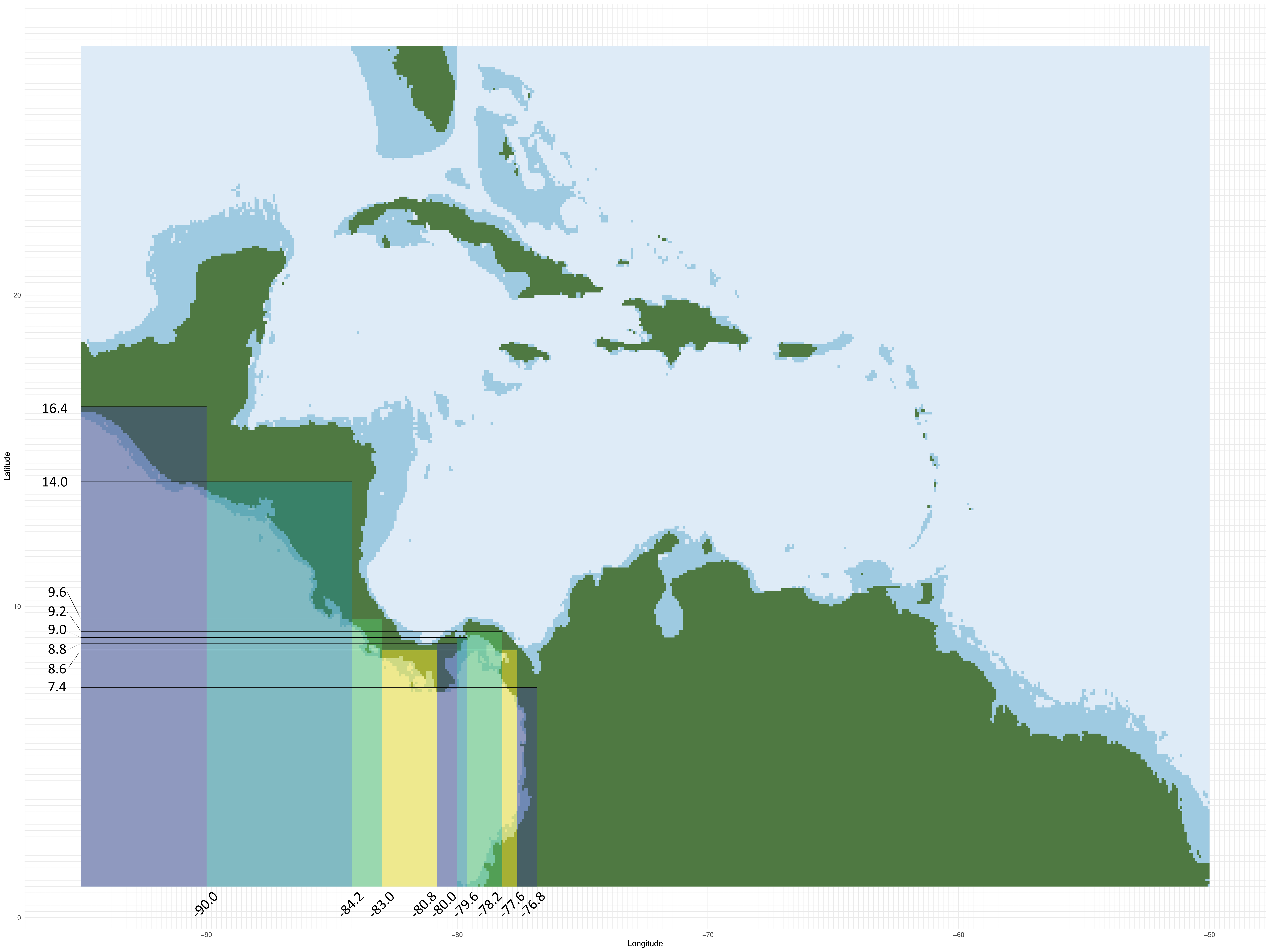


**Figure S1.** Maps showing rectangles used to determine whether a grid point was located in the Pacific or Caribbean basin. Grid points with latitudinal and longitudinal coordinates that fell within these coloured rectangles were designated ‘Pacific’.


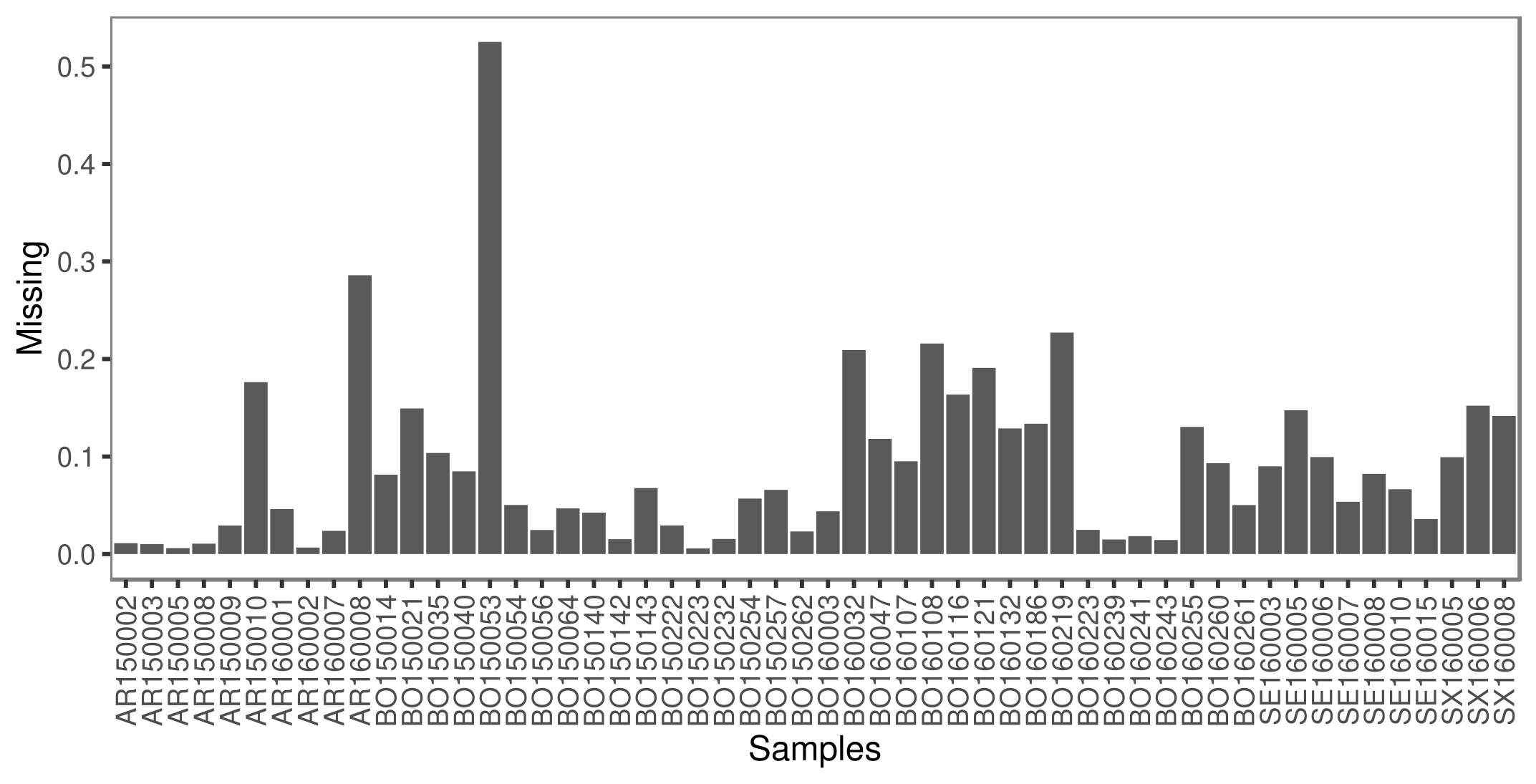


**Figure S2.** The amount of missing data per sample. Individual ‘BO150053’ was excluded from downstream analyses due to a high amount of missing data (>0.5).


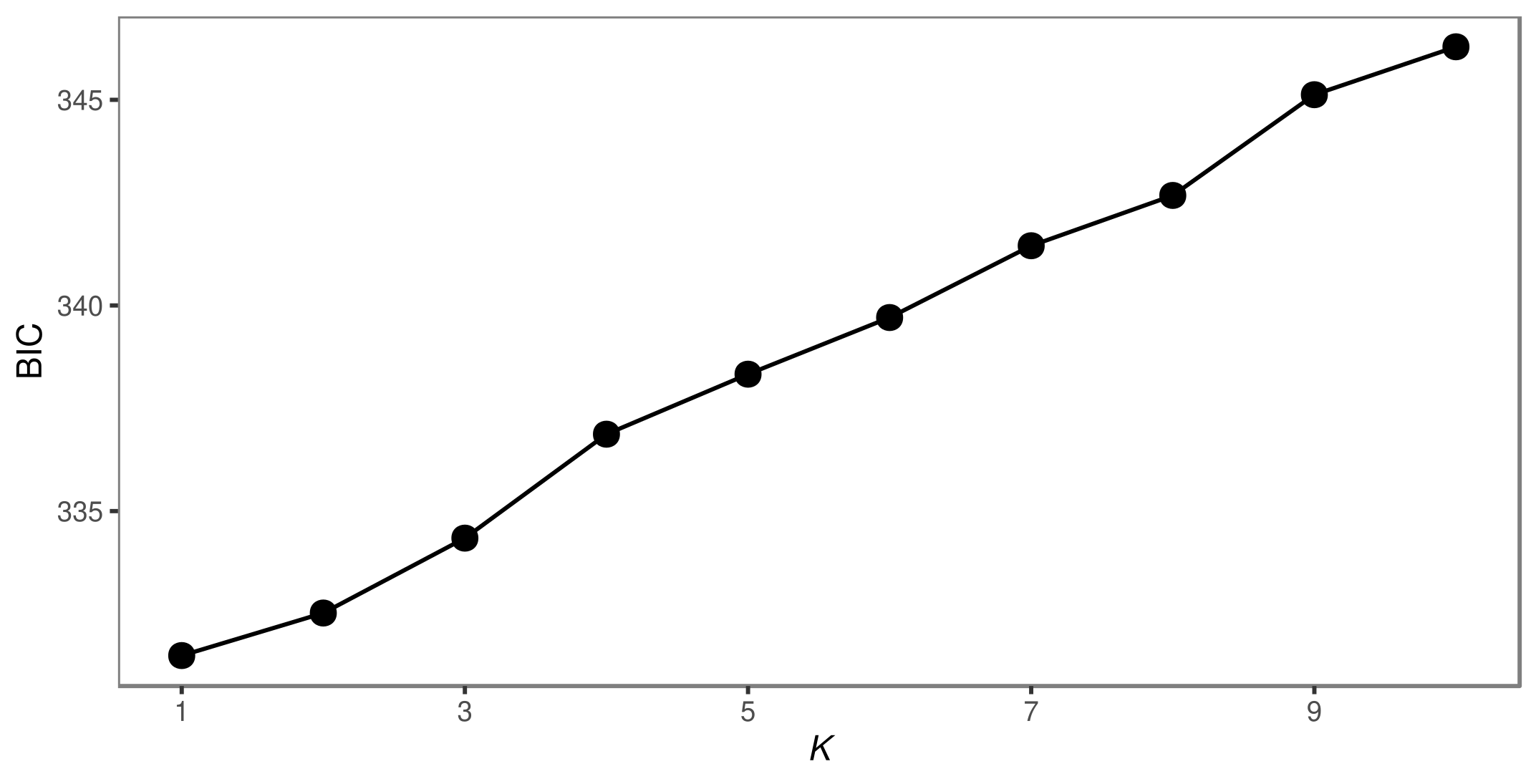


**Figure S3.** Plot of the Bayesian Information Criterion (BIC) versus the number of clusters *K* estimated using the *find.clusters()* function from the *adegenet* R package.


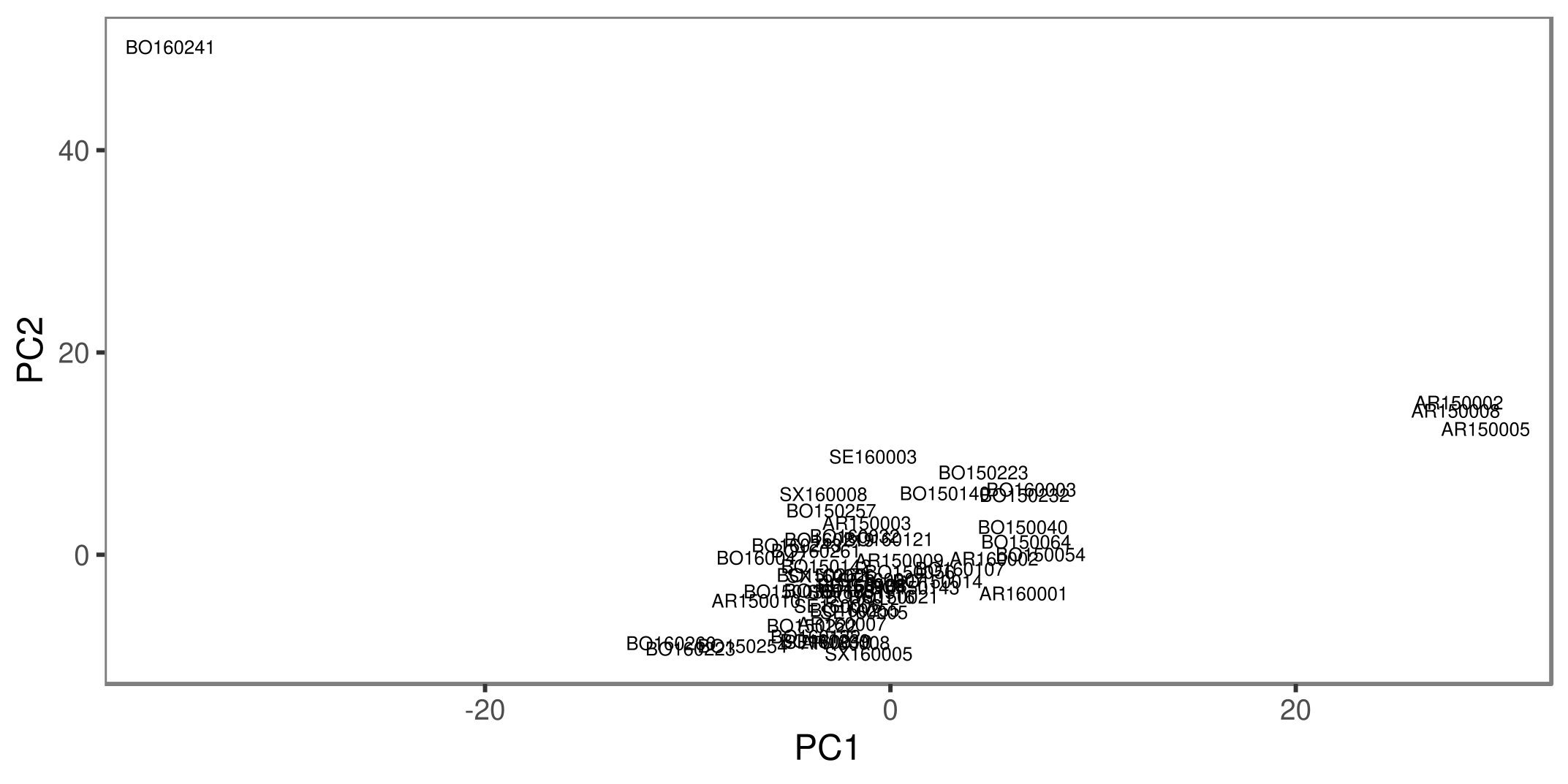


**Figure S4.** Plot of the first two principal components with individual sample labels estimated from the data including putative related individuals (*N* = 53).


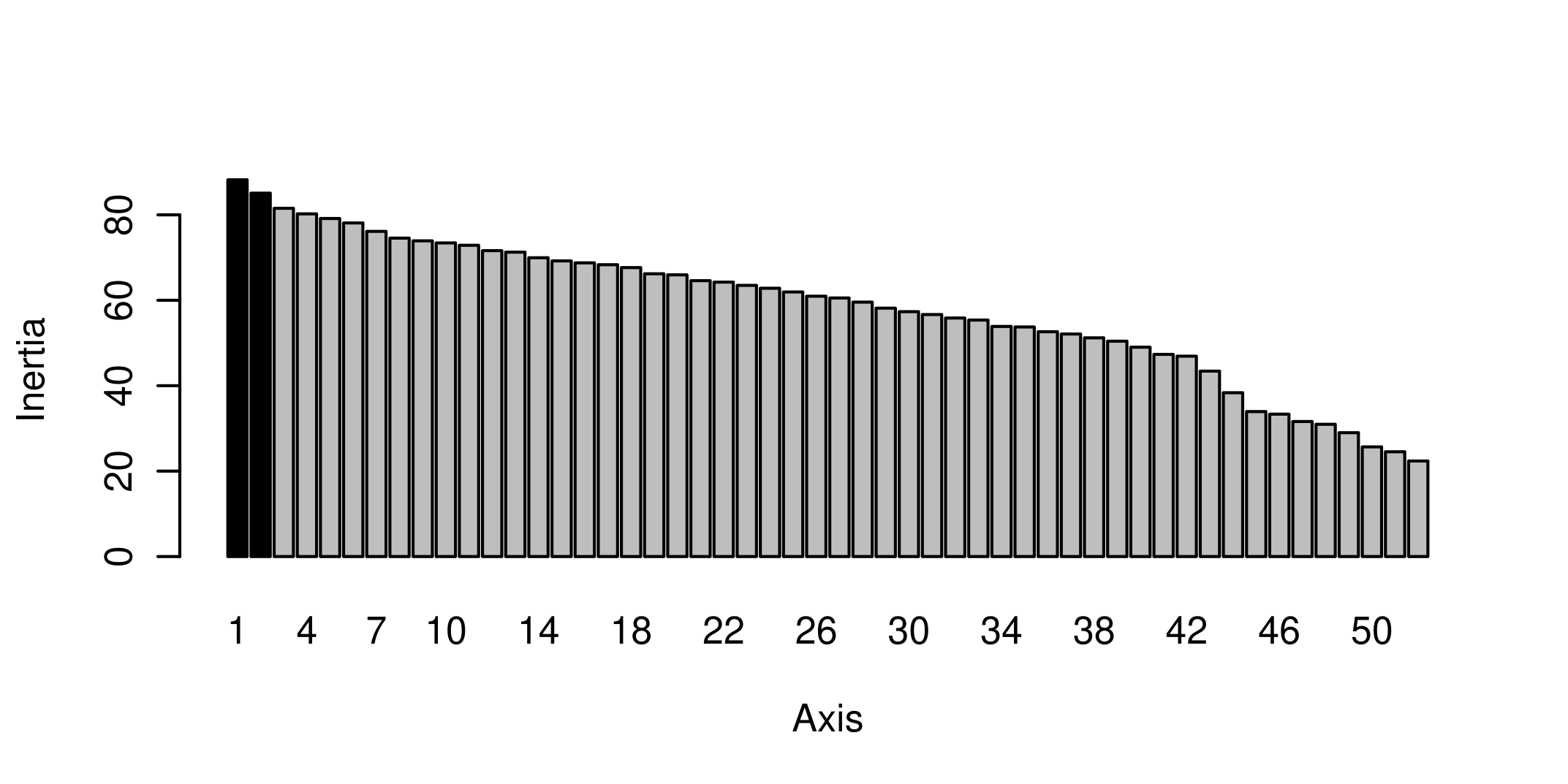


**Figure S5.** The distribution of total inertia across principal axes (data including putative related individuals; *N* = 53). The bars denote the eigenvalues associated with each principal component.


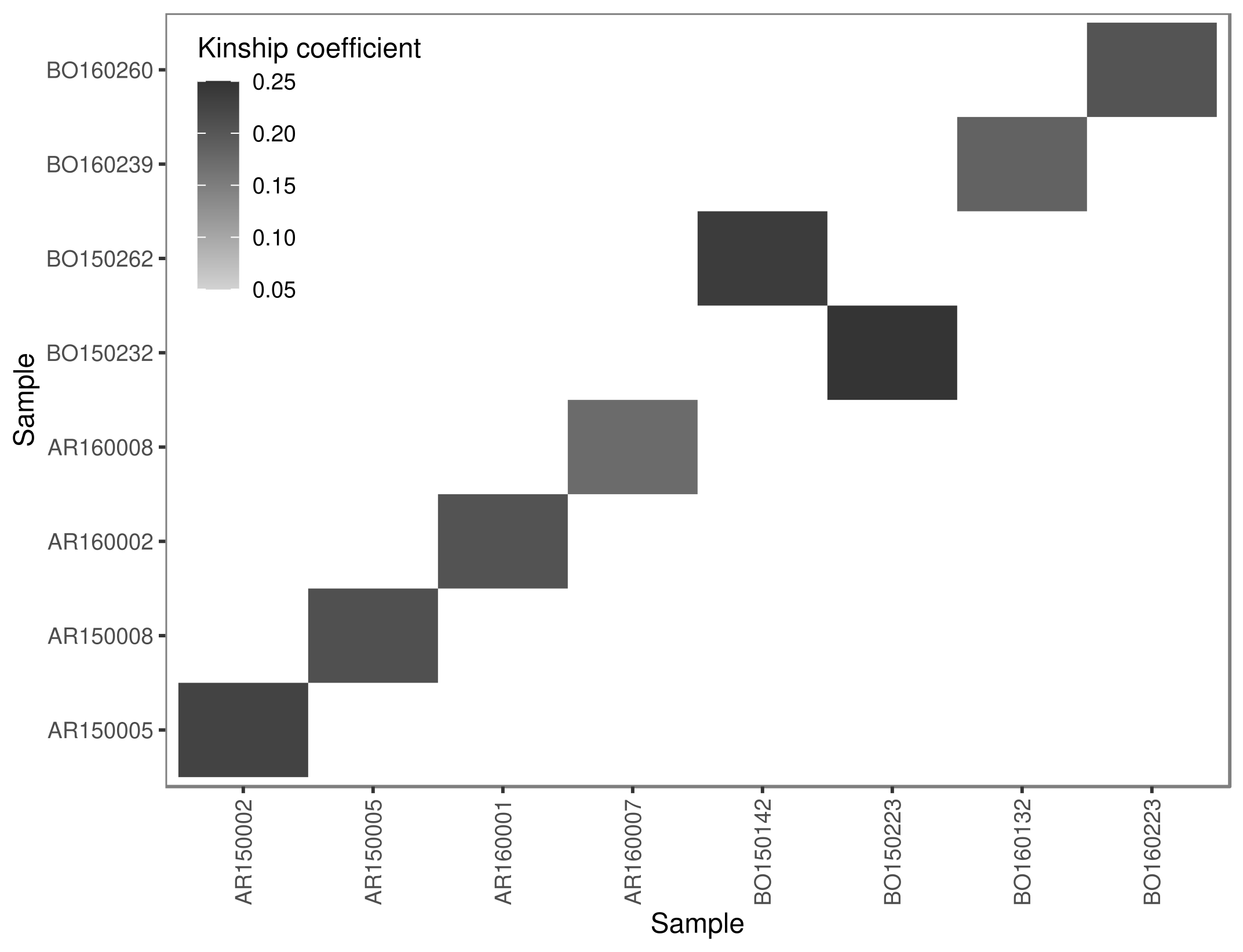


**Figure S6.** A matrix of estimated pairwise kinship coefficients.


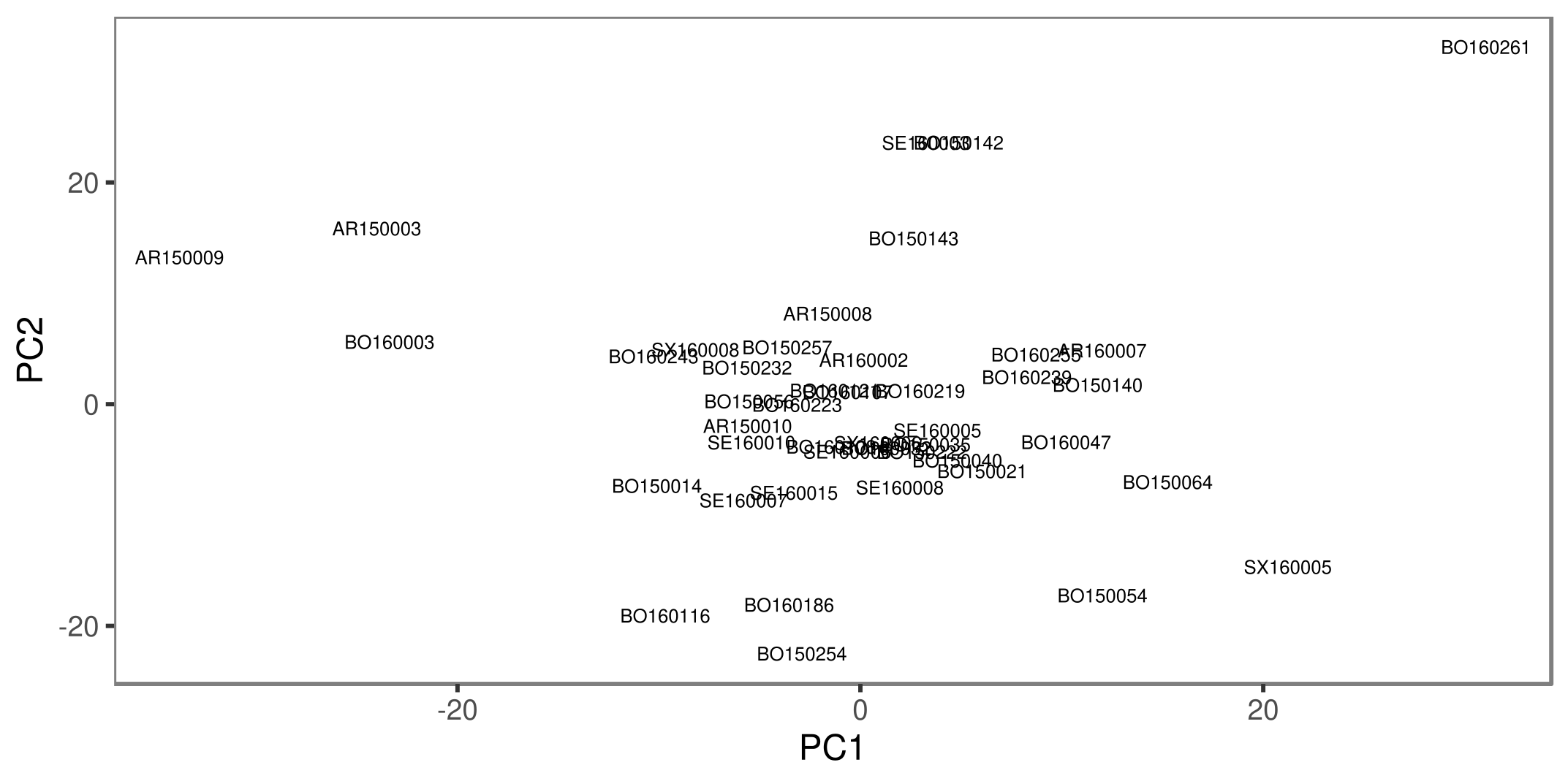


**Figure S7.** Plot of the first two principal components with individual sample labels estimated from the data excluding putative related individuals (*N* = 44).


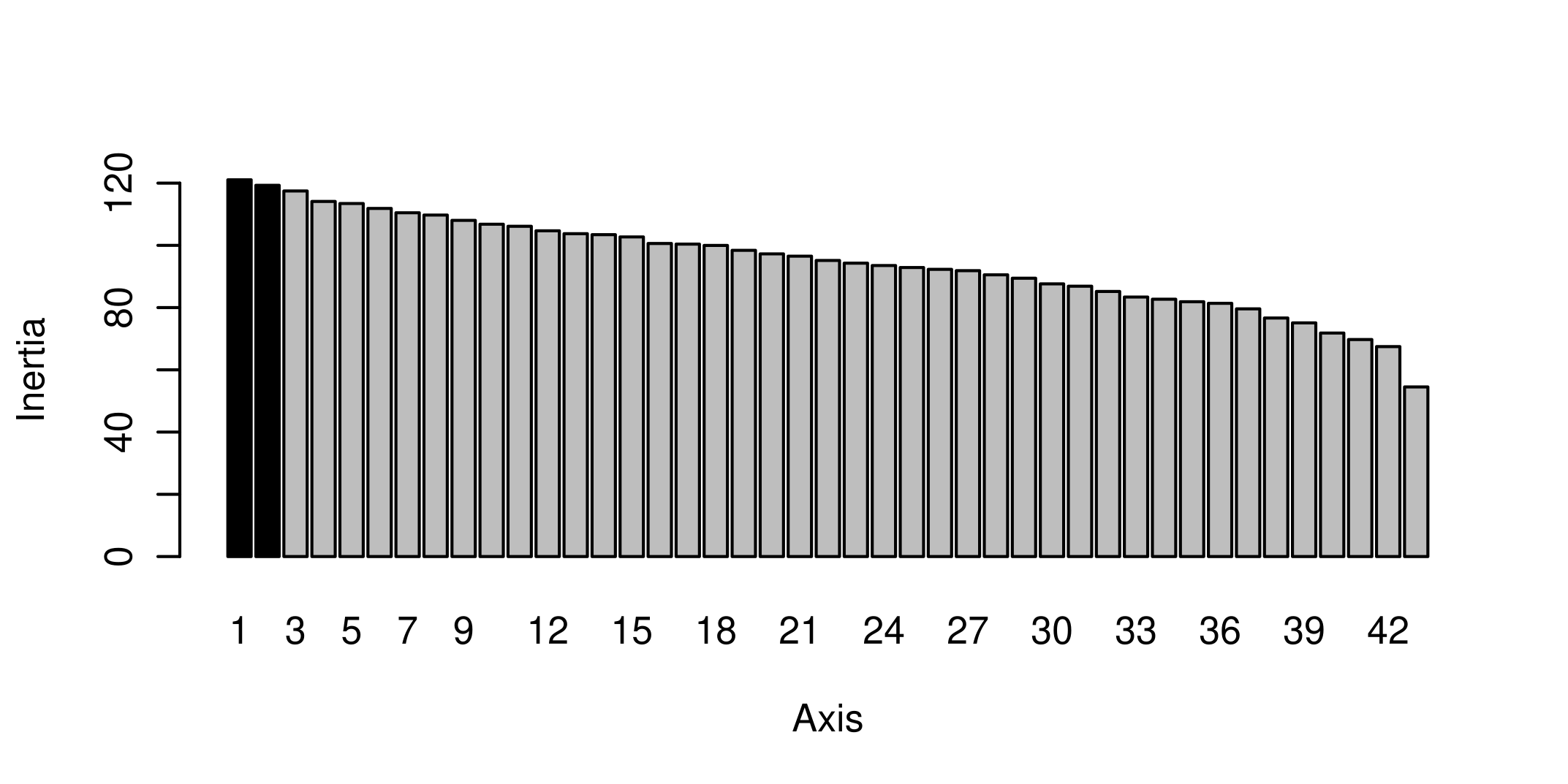


**Figure S8.** The distribution of total inertia across principal axes (data excluding putative related individuals; *N* = 44). The bars denote the eigenvalues associated with each principal component.


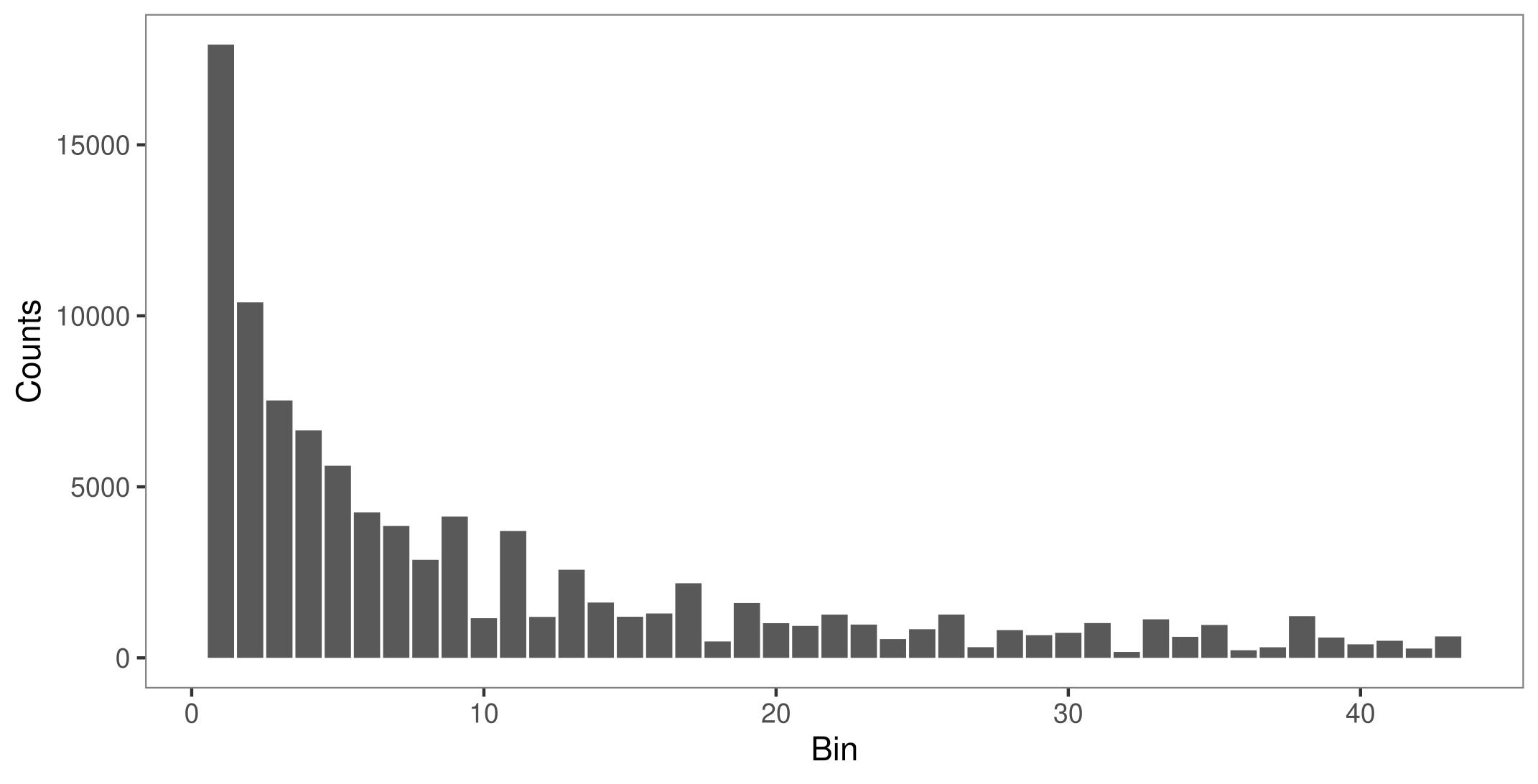


**Figure S9.** Folded site frequency spectrum estimated from the data excluding putative related individuals (*N* = 44 individuals).


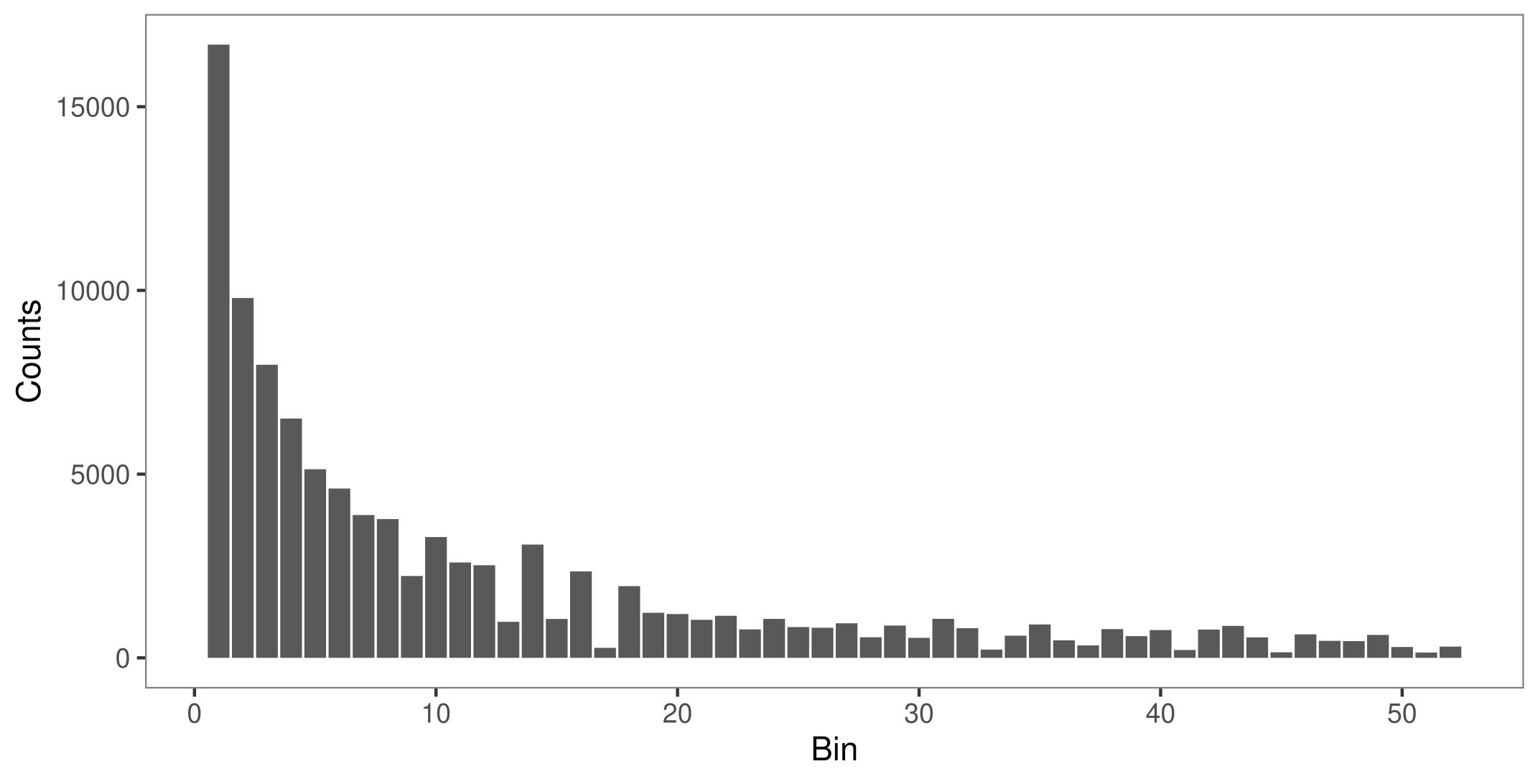


**Figure S10.** Folded site frequency spectrum estimated from the data including putative related individuals (*N* = 53 individuals).


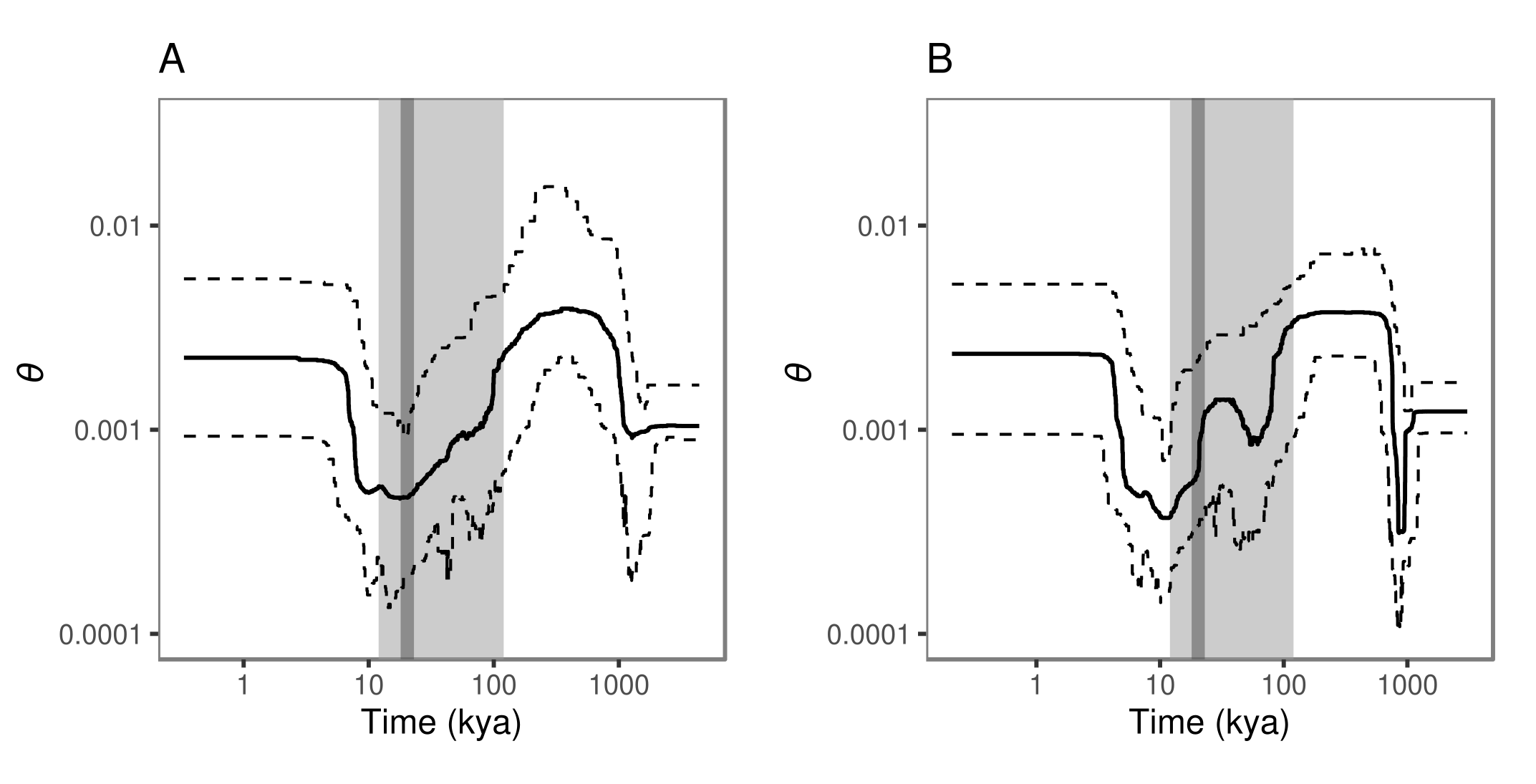


**Figure S11**. Median (solid line) and 95% confidence interval (dashed lines) of genetic diversity (*θ*) through time (kya) estimated from the data including putative related individuals (*N* = 53 individuals). Results are shown for (A) *μ* = 7.9 x 10^-9^ and (B) *μ* = 1.2 x 10^-8^ substitutions per site per generation. The approximate timing of the last glacial cycle (120 – 14 kya) is indicated in light grey shading. The LGM (26 – 19 kya) is indicated by a dark grey shaded bar.


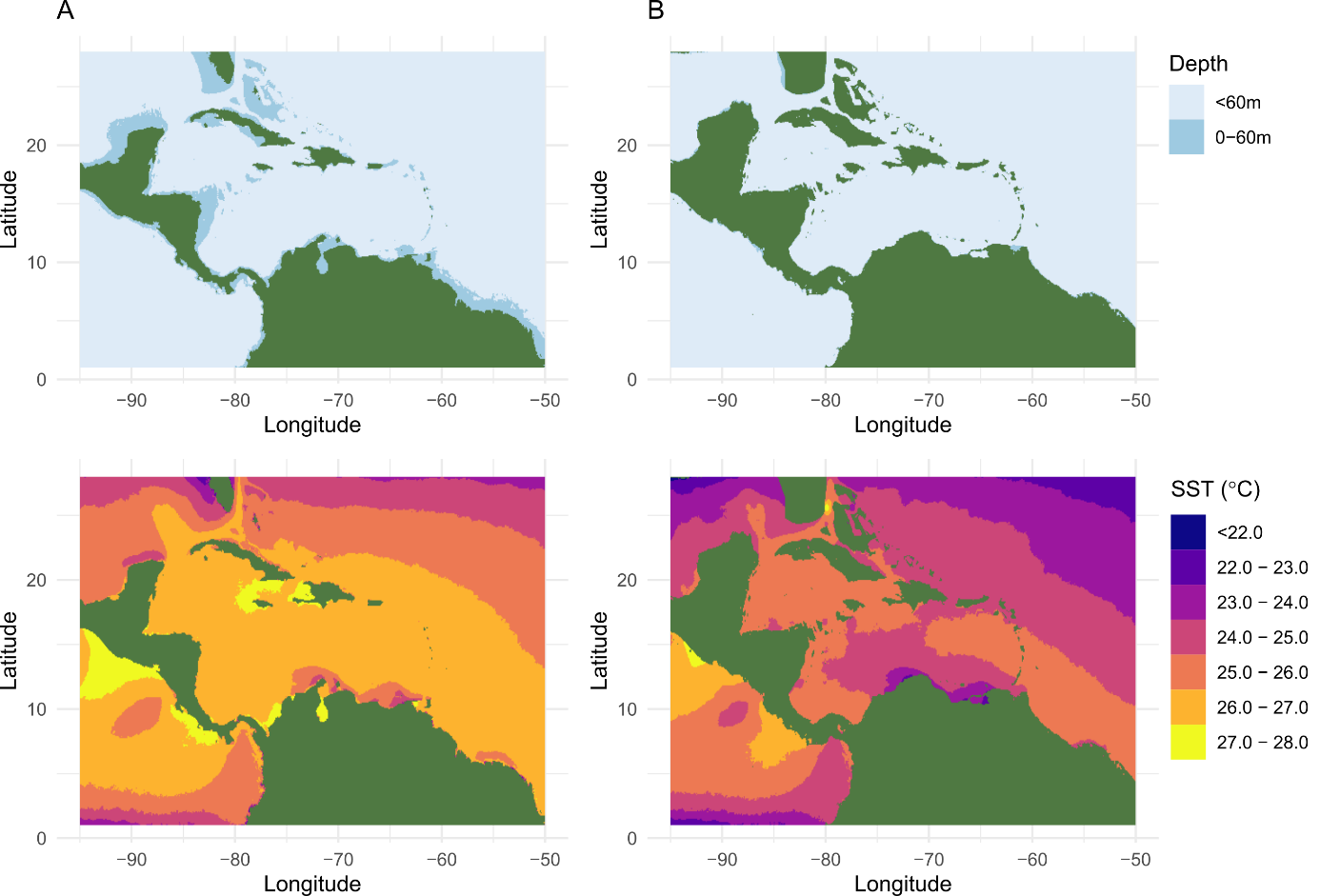


**Figure S12.** The distribution of shallow marine habitat (depth 0-60m; top panels) and mean annual sea surface temperature (SST in degrees Celsius, °C; bottom panels) during the (A) present and (B) Last Glacial Maximum (21 kya).

**Table S1.** Sample information, barcodes and adapters used in library preparation,summary of read statistics and alignment rate per sample (continued on next page).

**Table S1.** Sample information, barcodes and adapters used in library preparation, summary of read statistics and alignment rate per sample (continued from previous page).

**Table S2.** Functional annotation of SNPs according to genomic region, as well as predicted impacts and annotation descriptions (SNPEFF manual; Cingolani et al. 2012). Most SNPs were associated with intronic and intergenic regions, while a smaller proportion of SNPs were found to be downstream or upstream (within 5,000bp) of predicted gene regions. Note that SNPs can be associated with multiple regions. The predicted impacts were primarily classified as modifier (i.e., non-coding variants, variants of non-coding genes, no evidence of impact), with low impacts (i.e., likely harmless, unlikely to change protein functionality) for SNPs associated with splice site regions.

**Table S3.** The estimated marginal likelihoods for each explored value of the number of clusters K in the FASTSTRUCTURE analysis estimated from the data including putative related individuals (*N* = 53) and the data excluding putative related individuals (*N* = 44). An evaluation of the optimal model complexity using the *chooseK.py* script provided with FASTSTRUCTURE suggested the data was best described by a single cluster (i.e. *K* = 1) for both *N* = 53 and *N* = 44.

**Table S4.** Shallow marine habitat area (SMHA; measured as the number of grid points with depth 0-60m) and the relative amount of shallow marine habitat availability compared to the present (RSMHA). Estimates of SMHA and RSMHA obtained using a minimum sea surface temperature of 20 °C within grid points are shown within the parentheses.

**Table S5.** Correlation coefficients (measured as Pearson’s *r*) between median *θ* and 1) the relative amount of shallow marine habitat availability compared to the present (RSMHA), 2) the mean global surface temperature anomaly (SAT) in degrees Celsius (°C) and 3) the mean global sea level anomaly (SLA) for *μ* = 7.9 x 10^-9^ substitutions per site per generation (*μ*_L_) and *μ* = 1.2 x 10^-8^ substitutions per site per generation (*μ*_H_). Results are shown for different time sampling intervals (Δ) ranging between 1,000 and 5,000 years. A sampling interval of 1,000 indicates every 1000^th^ row was included (i.e., rows 1000, 2000, 3000 , … ).

**Table S6.** Values of the Aikaike information criterion (AIC), Bayesian information criterion (BIC), log-likelihood (LogL), model rankings and model coefficients (values, standard errors [SE] and associated *P*-values) for each model evaluated using generalized least squares, with genetic diversity (i.e., median *θ*) estimated using (A) *μ* = 7.9 x 10^-9^ and (B) *μ* = 1.2 x 10^-8^ substitutions per site per generation as response variable.
